# Isosulfan Blue and Anaphylaxis

**DOI:** 10.31486/toj.20.0162

**Published:** 2021

**Authors:** Abin Sajan, Daniel W. Griepp, Hazim Hakmi, Amir H. Sohail, Jackson Hunt, Michelle Wolf, Zhanna Logman

**Affiliations:** ^1^ Department of Surgery, NYU Langone Hospital–Long Island, Mineola, NY; ^2^ Department of Radiology, Columbia University Irving Medical Center, New York, NY; ^3^ New York Institute of Technology College of Osteopathic Medicine, Old Westbury, NY

**Keywords:** *Anaphylaxis*, *contrast*, *isosulfan blue*

## Abstract

**Background:** Isosulfan blue dye, or Lymphazurin, is commonly used for sentinel lymph node biopsy during operative procedures for patients with breast cancer. Allergic reactions to Lymphazurin have been reported, ranging from mild dermatologic reactions to severe anaphylaxis.

**Case Series:** We report 2 patients who experienced allergic reaction to Lymphazurin while admitted to our service. We also conducted a literature search for similar cases using national databases. Included studies were limited to retrospective studies, case series, or case reports. Patient characteristics, reaction observed, and hospital course were extracted. Of the patients we report, both had grade 3 anaphylactic reactions requiring vasopressors to achieve hemodynamic stability. One patient required intensive care unit monitoring for 18 hours, and the other required overnight monitoring in the postanesthesia care unit. The literature revealed 29 studies reporting 108 patients with confirmed allergic reactions to Lymphazurin. Including the 2 patients in this series (total study n=110), most reactions were grade 3 (57/110, 51.8%), followed by grade 1 (40/110, 36.4%) and grade 2 (13/110, 11.8%). Among the patients who had individual hospital course reported (n=34), 23 patients required admission to the surgical intensive care unit. Of studies that reported cancellation or progression of surgery after the reaction, the surgical procedure was canceled for 12 of 26 patients (46.1%).

**Conclusion:** Although severe anaphylactic reactions are more commonly reported, mild reactions occur more frequently but are likely underreported. Although no fatalities were reported in the cases included in this review, anaphylactic reactions to Lymphazurin pose significant risks. Operating room personnel should be familiar with potential reactions to recognize and treat them early.

## INTRODUCTION

Isosulfan blue dye or Lymphazurin, an isomer of patent blue dye, is an integral component of lymphangiography in sentinel lymph node biopsy (SLNB).^1-3^ Typically injected preprocedurally, isosulfan blue dye is selectively absorbed by the lymphatics and aids in visualizing the lymphatic channels. Allergic reactions to isosulfan blue dye have been previously reported, with an incidence of 0.6% to 2.5%.^4,5^ Allergic reactions can range from localized skin changes to life-threatening multiorgan failure.^6,7^ The prevalence of SLNB with isosulfan blue dye has extended outside breast cancer, with new indications in melanoma and neoplasms of the bladder, cervix, and uterus.^2^ As the use of isosulfan blue dye has increased, reports of associated adverse reactions have increased. However, synthesis of previously reported allergic reactions to isosulfan blue dye is limited, particularly with regard to the effect on hospital course, such as cancellation of surgery or time spent in the intensive care unit.

We report 2 cases of severe anaphylaxis to isosulfan blue dye and present a review of reported cases of allergic reactions to isosulfan blue dye.

## CASE SERIES

### Case 1

A 46-year-old female with high-grade comedo necrosis ductal carcinoma in situ of the left breast presented for elective lumpectomy and SLNB. She had a history of hypertension and ovarian cysts with no previously reported allergic reactions. On the day of the surgery, 5 mL Lymphazurin was injected intradermally into the breast tissue surrounding the site of carcinoma. The surgical site was prepped with Betadine (povidone-iodine), and anesthesia was induced with 100 μg intramuscular fentanyl, 2 mg intravenous midazolam, and 200 mg propofol. A laryngeal mask airway (LMA) was inserted, and the patient was administered 1 g cefazolin and 30 mg lidocaine HCl 1% for local anesthesia.

Five minutes after administering Lymphazurin and before the first incision, the patient became tachycardic to the 120/min range, and systolic blood pressure dropped to 75 mmHg. Shortly after, the patient appeared flushed with swollen distal extremities, suggestive of an anaphylactic reaction. Her systolic pressure was mildly responsive to 2 doses of phenylephrine 100 μg and reached as low as 60 mmHg approximately 10 minutes after the initial reaction. She was given 100 μg epinephrine, as well as injections of 50 mg diphenhydramine and 100 mg hydrocortisone. Given her compromised hemodynamic status, the patient was successfully intubated with an endotracheal tube, and the planned procedure was canceled. Table 1 summarizes the pharmacologic agents administered, including those given after the decision was made to cancel the surgery.

**Table 1.** Pharmacologic Agents Administered

Case 1	Quantity	Case 2	Quantity
Midazolam 2 mg IV	2	Midazolam 2 mg IV	2
Fentanyl 100 μg IM	1	Fentanyl 50 μg IV	1
Lidocaine HCl 1% 30 mg subq	1	Lidocaine HCl 1% 40 mg subq	1
Propofol 200 mg IV	1	Propofol 200 mg IV	1
Cefazolin 1 g IV	1		
Phenylephrine 100 μg IV	2	Phenylephrine 200 μg IV	4
Hydrocortisone 100 mg IV	1	Hydrocortisone 100 mg IV	1
Diphenhydramine 50 mg IV	1	Diphenhydramine 50 mg IV	1
Ephedrine 10 mg IV	2	Ephedrine 20 mg IV	2
Epinephrine 100 μg IV	3	Epinephrine 30 μg IV	3

IM, intramuscular; IV, intravenous; subq, subcutaneous.

The patient was transferred to the surgical intensive care unit (SICU) and closely monitored. She remained intubated overnight and was weaned off the vasopressors by the morning. On hospital day 1, she was extubated and downgraded without issues. She was discharged on hospital day 2 with 20 mg famotidine and a 20 mg hydrocortisone taper. Despite multiple attempts to contact the patient, she was lost to follow-up and did not undergo allergy testing.

### Case 2

A 51-year-old female with high-grade comedo necrosis ductal carcinoma in situ of the right breast presented for elective excision of right breast calcification and SLNB. She had a history of allergic reaction (mild pruritus) to apples, peaches, plums, and cherries. On the day of surgery, 5 mL Lymphazurin was injected, and she was prepped with Betadine (povidone-iodine). An LMA was inserted after administering 2 mg midazolam, 50 μg fentanyl, and 200 mg propofol.

After local anesthesia (40 mg lidocaine HCl 1%) was administered, the SLNB was successfully completed in 15 minutes. Approximately 25 minutes after injecting isosulfan blue dye during the mass excision portion of the case, the patient's blood pressure dropped to 50/30 mmHg with associated bradycardia in the 60/min range. Anaphylactic reaction was suspected given the patient's facial flushing and swelling of the upper extremities. During the next 30 minutes, the patient received multiple vasopressor infusions, including phenylephrine 200 μg, epinephrine 30 μg, and ephedrine 20 mg. The patient also required administration of additional pharmacologic agents that are listed in Table 1. The patient was intubated with an endotracheal tube because of prolonged hemodynamic instability. Approximately 90 minutes after the initial injection, systolic pressure sustained in the 100 mmHg range, and the case was completed successfully. The patient was transferred to the postanesthesia care unit (PACU) and monitored overnight. She was stable overnight and discharged the following day from the PACU with no complaints. Outpatient workup isolated the allergic reaction to isosulfan blue dye and eliminated allergies to any anesthetic and analgesic agents. Table 2 summarizes the outpatient workup by the allergist confirming allergy to isosulfan blue dye.

**Table 2.** Outpatient Allergy Skin Testing Results for Case 2

Product	Prick, mm	Intradermal, mm	Result
Histamine	4,8	10,21	Positive control
Diluent control	0,0	0,0	Negative control
Midazolam, 0.5 mg/mL	0,0		Negative
Midazolam, 1:100		0,0	Negative
Midazolam, 1:10		0,0	Negative
Fentanyl, 0.05 mg/mL	0,0		Negative
Fentanyl, 1:1,000		0,0	Negative
Fentanyl, 1:100		0,0	Negative
Propofol, 10 mg/mL	0,0		Negative
Propofol, 1:100		0,0	Negative
Propofol, 1:10		0,0	Negative
Cefazolin, 330 mg/mL	0,0		Negative
Cefazolin, 1:100		0,0	Negative
Cefazolin, 1:10		0,0	Negative
PRE-PEN (benzylpenicilloyl polylysine: 10,000 U/mL)	0,0	0,0	Negative
Penicillin G, (benzylpenicillin: 10,000 U/mL)	0,0	0,0	Negative
Lidocaine 2%, 20 mg/mL	0,0		Negative
Lidocaine, 1:100		0,0	Negative
^a^Isosulfan blue 1%, 10 mg/mL	6,8		Positive
^a^Isosulfan blue 1%, 10 mg/mL	5,5		Positive
Isosulfan blue, 1:10,000	0,0		Negative
Isosulfan blue, 1:1,000	11,22		Positive

Note: Table shows negative skin testing to PRE-PEN and Penicillin G via intradermal and percutaneous methods with appropriate positive and negative controls. The patient tested negative to midazolam, fentanyl, propofol, cefazolin, and lidocaine but positive to isosulfan blue.

^a^Test was repeated to confirm reaction to isosulfan blue.

## DISCUSSION

Reports of allergic reactions to patent blue dye (parent molecule of isosulfan blue dye) trace back to 1966 when Kopp described 2 cases of anaphylaxis during lymphangiography.^8^ The US Food and Drug Administration approved isosulfan blue dye for lymphatic mapping in 1982, and a case report in the same year by Rubis et al was the first to report an allergic reaction specific to Lymphazurin.^9^ The reported cases have increased over the years, ranging from self-resolving erythema or urticaria to complicated cases of cardiovascular or respiratory collapse requiring SICU monitoring.^5,10,11^ The current estimated incidence of adverse reactions to isosulfan blue dye is as high as 2.5%.^4,11^

The pathophysiology of adverse reactions to isosulfan blue dye is not well understood. The anaphylactic reaction involves development of immunoglobulin E antibodies against foreign material.^10^ The antigen causes cross-linking and degranulation of mast cells, resulting in the release of histamine and other vasoactive mediators of anaphylaxis.^4^ Other postulated mechanisms include disorders in the arachidonic acid metabolism, direct activation of mast cells, and idiopathic anaphylaxis.^12,13^

Montgomery et al classified the range of adverse reactions secondary to isosulfan blue dye into 3 grades.^14^ Grade 1 is the simplest reaction and includes urticaria, pruritus, and at times, a rash with or without hives. Grade 2 involves transient hypotension not requiring vasopressors, and grade 3 involves hypotension requiring vasopressors. In the present case series, both patients had grade 3 reactions that required vasopressors and close hemodynamic monitoring.

Review of the literature using national databases (Medline, Embase, and Cochrane) revealed 29 studies reporting 108 patients who had confirmed reported cases of adverse events to isosulfan blue dye.^1-7,10,11,14-33^ Tables 3 and 4 provide a summary of characteristics, reactions observed, and clinical course of the patients in studies included in this review. Including the 2 patients in the present series (n=110), most patients had a grade 3 (57/110, 51.8%) reaction, followed by grade 1 (40/110, 36.4%) and grade 2 (13/110, 11.8%). Among the patients for whom hospital course was reported (n=34), 23 known patients were admitted to the SICU. Additionally, the planned surgery was canceled for 12 of 26 patients for whom these data were available, but the status of the planned procedure was unknown for 84 patients. In our series, the first patient required SICU monitoring and was discharged on the second hospital day, while the second patient had a less severe reaction, recovered more favorably, was able to complete the planned surgery, and only required overnight PACU monitoring prior to discharge.

**Table 3.** Characteristics of Studies Included in the Review

Study	N	Age, years, Sex	Dose	Grade 1	Grade 2	Grade 3	Time From Administration to Reaction	Surgery Canceled	Time in SICU
Longnecker et al, 1985^17^	1	N/R	N/R			1	1 min	N/R	24 h
Leong et al, 2000^18^	3	38, M	4.8 mL			1	30 min	Yes	36 h
		66, F	4.7 mL			1	15 min	No	None
		81, F	4.8 mL			1	15 min	Yes	48 h
Lyew et al, 2000^5^	1	48, F	5 mL			1	5 min	No	18 h
Cimmino et al, 2001^11^	5	22, F	3 mL			1	10 min	N/R	Yes, time N/R
		72, M	3 mL			1	8 min	N/R	Yes, time N/R
		50, F	3 mL			1	40 min	N/R	N/R
		47, F	5 mL		1		30-40 min	N/R	N/R
		47, F	5 mL		1		30-40 min	N/R	N/R
Albo et al, 2001^4^	7	75, F	5 mL			1	20 min	N/R	48 h
		75, F	5 mL			1	15 min	N/R	24 h
		50, F	5 mL			1	15 min	N/R	24 h
		47, F	5 mL			1	15 min	N/R	24 h
		65, F	5 mL			1	30 min	N/R	48 h
		53, F	5 mL			1	20 min	N/R	72 h
		53, F	5 mL			1	30 min	N/R	24 h
Kuerer et al, 2001^19^	1	75, F	5 mL			1	40 min	No	N/R
Krouse and Schwarz, 2001^20^	1	63, F	4 mL		1		N/R	No	N/R
Sadiq et al, 2001^21^	2	52, F	2 mL		1		45 min	No	None
		57, F	2 mL		1		25 min	Yes	None
Kuerer et al, 2001^22^	1	52, F	5 mL			1	N/R	Yes	N/R
Giménez et al, 2001^23^	2	48, F	4 mL			1	5 min	No	N/R
		60, F	2 mL			1	5 min	No	None
Laurie et al, 2002^10^	2	60, F	5 mL			1	5 min	Yes	48 h
		62, F	5 mL			1	40 min	Yes	None
Montgomery et al, 2002^14^	39	N/R	N/R	27	3	9	44 min (mean)	N/R	N/R
Efron et al, 2002^24^	1	54, F	5 mL			1	10 min	No	24 h
Stefanutto et al, 2002^25^	1	N/R	N/R			1	N/R	N/R	N/R
Sprung et al, 2003^26^	1	53, F	4 mL			1	1 min	Yes	N/R
Raut et al, 2004^27^	3	N/R	5 mL	3			N/R	N/R	N/R
Sandhu et al, 2005^6^	1	45, F	5 mL			1	10 min	Yes	48 h
Raut et al, 2005^15^	4	73, F	5 mL	1			105 min	N/R	None
		62, F	5 mL	1			45 min	N/R	None
		53, F	5 mL	1			75 min	N/R	None
		58, F	5 mL	1			10 min	N/R	None
Amr et al, 2005^16^	7	N/R	N/R	6		1	N/R	N/R	N/R
Komenaka et al, 2005^28^	3	N/R	N/R		3		23 min (mean)	N/R	N/R
Saft and Sarap, 2007^29^	1	54, F	5 mL			1	20 min	Yes	N/R
Kaufman et al, 2008^2^	2	62, F	5 mL			1	30 min	N/R	24 h
		77, M	1.8 mL			1	1 min	N/R	24 h
Liang and Carson, 2008^7^	1	48, F	5 mL			1	15 min	Yes	36 h
O’Sullivan and Morrow, 2008^30^	1	77, F	8 mL		1		N/A	No	None
Cinar et al, 2012^31^	1	65, F	5 mL			1	30 sec	No	2 h
Haque and Nossaman, 2012^1^	2	83, F	4 mL		1		N/R	No	N/R
		62, F	5 mL			1	Within minutes	No	N/R
Reed et al, 2014^32^	1	44, F	N/R			1	20 min	No	N/R
Ortiz et al, 2015^33^	1	82, F	1 mL			1	15 min	Yes	48 h
Wang et al, 2018^3^	12	N/R	N/R			12	N/R	N/R	Yes, time N/R
Present Study	2	46, F	5 mL			1	5 min	Yes	18 h
		51, F	5 mL			1	25 min	No	None

N/R, not reported; SICU, surgical intensive care unit.

**Table 4.** Summary of Clinical Course of Patients Including the Present Series

Variable	Value
Demographics	
Total patients, n	110
Age, years, mean (range); n=44	58.3 (22-83)
Age not available, n (%)	66/110 (60)
Male, n (%)	3/44 (6.8)
Female, n (%)	41/44 (93.2)
Reaction, n (%)	
Grade 1–Generalized swelling, urticaria, mild rash	40/110 (36.4)
Grade 2–Hypotension, vasopressors not given	13/110 (11.8)
Grade 3–Hypotension, vasopressors given	57/110 (51.8)
Dosage of isosulfan blue, mL, median (range); n=44	5 (1.0-8.0)
Administration to reaction time, min, mean (range); n=42	23.28 (0.5-105)
Surgery status, n (%); n=26	
Canceled	12/26 (46.1)
Not canceled	14/26 (53.8)
Not available	84/110 (76.4)
Surgical intensive care unit admission; n=34	
Yes, n (%)	23/34 (67.6)
Time in intensive care, h, mean (range)	33.1 (2-72)
No, n (%)	11/34 (32.4)
Not available, n (%)	76/110 (69.1)
Death	None

Given the potential for complex pharmacologic interactions among antibiotics and anesthetic agents administered during surgical procedures, isolating the reaction can be difficult.^15^ Although the temporal relationship provides a challenge, the rate of allergic reactions to antibiotics and anesthetic agents is considered significantly smaller than the rate of allergic reactions to isosulfan blue dye. For example, cefazolin allergy has been reported in 1/17,000 (0.006%) cases compared to the reported 0.6% to 2.5% of isosulfan blue dye allergies.^4,5,11^ Additionally, most patients have been previously exposed to beta-lactam antibiotics.^4^ One patient in our series underwent outpatient allergy profile testing to rule out reactions to the antibiotics and anesthetic agents used during surgery (Table 2).

Severe allergic reactions to isosulfan blue dye can extend hospital stay, and the associated intensive care poses a significant financial and mental burden on patients. Studies have previously explored the alternatives to isosulfan blue dye, such as fluorescent dye or methylene blue; however, similar reactions have been reported.^34^ Skin testing prior to the procedure or preprocedural steroids or antihistamines are other options to avoid anaphylaxis in this setting, especially in high-risk patients with similar allergies or history of asthma. The benefits of these interventions should be weighed against risks or health care costs through future prospective studies.

The true incidence of isosulfan blue dye allergic reaction is likely higher than the reported 0.6% to 2.5%. Despite the range of allergic reaction—from skin changes to anaphylaxis—the literature principally reports anaphylaxis-type reactions requiring SICU level of care. Selective reporting of more severe reactions introduces selection bias as the patients with the most severe reactions who warrant higher levels of care are more likely to be reported than patients with less severe reactions. Of the studies included in this review, 19 of 29 studies reported only patients who had experienced grade 3 reactions. The true incidence of total isosulfan blue dye allergic reaction (regardless of grade) is likely higher than the cases identified in the literature would suggest; the percentage of anaphylaxis/shock grade 3 among all patients with isosulfan blue dye allergy likely remains low.

## CONCLUSION

The use of isosulfan blue dye is a valuable technique in lymph node biopsy; however, isosulfan blue dye is associated with anaphylactic reactions and the consequences may be serious in some cases, as summarized in this review. These cases underscore the importance for operating room personnel to be familiar with such potential reactions so they can recognize and effectively treat them early in acute care settings.

## Notes

*This article meets the Accreditation Council for Graduate Medical Education and the American Board of Medical Specialties Maintenance of Certification competencies for Patient Care and Medical Knowledge.*
